# *Cananga odorata* (Ylang-Ylang) Essential Oil Containing Nanoemulgel for the Topical Treatment of Scalp Psoriasis and Dandruff

**DOI:** 10.3390/gels10050303

**Published:** 2024-04-30

**Authors:** Perwez Alam, Mohd Imran, Asad Ali, Haya Majid

**Affiliations:** 1 Department of Pharmacognosy, College of Pharmacy, King Saud University, P.O. Box 2457, Riyadh 11451, Saudi Arabia; 2 R&D Executive, Aimil Pharmaceuticals, New Delhi 110028, India; imranidrisi00786@gmail.com; 3 Department of Pharmaceutics, School of Pharmaceutical Education and Research, Jamia Hamdard, New Delhi 110062, India; asad97111@gmail.com; 4 Department of Translational and Clinical Research, School of Chemical and Life Sciences, Jamia Hamdard, New Delhi 110062, India; hayamajid1.hm@gmail.com

**Keywords:** scalp psoriasis, dandruff, *Cananga odorata*, nanoemulgel, topical delivery, antimicrobial and antifungal activity

## Abstract

This research aimed to evaluate the efficacy of a nanoemulgel (NE) containing *Cananga odorata* (Ylang-Ylang) oil for managing scalp psoriasis and dandruff through various assessments. The study involved phytochemical screening, characterization, stability testing, in vivo performance evaluation, dermatokinetic analysis, central composite rotatable design (CCRD) optimization, in vitro release profiling, and antioxidant and antimicrobial activity assessment of the NE. The NE exhibited excellent stability and maintained physical parameters over a three-month period. In vivo studies showed no skin irritation, maintenance of skin pH (4.55 to 5.08), and improvement in skin hydration (18.09 to 41.28 AU) and sebum content (26.75 to 5.67 mg/cm^2^). Dermatokinetic analysis revealed higher skin retention of *C. odorata* in the NE (epidermis: 71.266 µg/cm^2^, dermis: 60.179 µg/cm^2^) compared to conventional formulations. CCRD optimization yielded NE formulations with the desired particle size (195.64 nm), entrapment efficiency (85.51%), and zeta potential (−20.59 mV). In vitro release studies indicated sustained release behavior, and antioxidant and antimicrobial properties were observed. This study demonstrates the stability, skin-friendliness, therapeutic benefits, and controlled release properties of the NE. The NE presents a promising option for various topical applications in treating bacterial and fungal diseases, potentially enhancing drug delivery and treatment outcomes in pharmaceuticals and cosmetics.

## 1. Introduction

Scalp psoriasis and dandruff are common dermatological conditions affecting a significant portion of the population worldwide. These conditions can cause scalp itching, flaking, inflammation, and discomfort, negatively impacting the quality of life [1]. Globally, psoriasis affects about 0.84% of the total population with 811 cases per 100,000 individuals [2]. Traditional treatments for scalp psoriasis and dandruff often involve the use of corticosteroids and antifungal agents, which may have side effects and limited efficacy [3]. Therefore, there is a need for alternative therapies that are safe, effective, and well-tolerated [4].

*Cananga odorata*, commonly known as Ylang-Ylang, is a tropical plant native to Southeast Asia. It is known for its aromatic properties and has traditionally been used with various therapeutic purposes, including treatment of skin disorders [5]. Ylang-Ylang essential oil extracted from the plant possesses antimicrobial, anti-inflammatory, and antioxidant properties, making it a potential candidate for treating scalp psoriasis and dandruff [6]. 

Nanoemulsions (NEs) have garnered significant attention in the field of topical formulations due to their unique properties and potential applications. These colloidal systems consist of small droplets of one immiscible liquid dispersed within another immiscible liquid, stabilized by surfactants or cosurfactants. The small droplet size in the range of approximately 20–1000 nm and large interfacial area of NEs offer advantages such as improved stability, enhanced drug solubility, controlled release, and increased bioavailability. These attributes make NEs promising candidates for delivering various active ingredients, including drugs, essential oils, and cosmeceuticals, to the skin [7]. Phytocompound-based nanoemulgels are increasingly studied for their potential in various therapeutic and cosmetic applications due to their ability to enhance the delivery and efficacy of bioactive compounds derived from plants. Nanoemulgel formulations improve the permeation behavior of phytocompounds across skin barriers, which improves drug distribution and absorption in topical applications, enhancing their therapeutic effectiveness [8]. Nanoemulgel formulations have been effectively used for delivering anti-inflammatory phytocompounds, enhancing their release behavior and improving conditions like arthritis and skin inflammation [9]. The formulation allows for a controlled and sustained release of phytocompounds, which is beneficial for maintaining the desired concentration of the drug over an extended period. This is particularly useful in treating chronic conditions. Nanoemulgels provide a non-invasive method of delivery, which is preferable for patients who are averse to injections or oral medications. This increases patient compliance and convenience [10].

Several in silico-based technologies have been applied to gather information on the mechanistic properties of nano-based topical formulations, which have shown efficient results [11,12,13,14]. The development of NE formulations requires an understanding of their physicochemical characteristics and their impact on skin compatibility, stability, and therapeutic effects. Characterization techniques such as droplet size analysis, polydispersity index (PDI) measurement, and zeta potential determination provide valuable insights into the stability and uniformity of NEs. Additionally, imaging techniques like transmission electron microscopy (TEM) offer visual confirmation of droplet size and distribution within the formulation [15]. Understanding the physicochemical characteristics of NEs is crucial for optimizing their formulation parameters and ensuring their suitability for topical applications. In addition to stability and formulation parameters, skin compatibility is a vital aspect to consider in topical formulations. Skin irritation potential, maintenance of the skin’s natural pH, and enhancement of skin hydration are key factors for evaluating the compatibility and efficacy of NEs. Skin irritation tests provide information on the potential adverse effects of NEs on the skin, while pH measurements determine the impact on the skin’s acid mantle [16]. Furthermore, the ability of NEs to improve skin hydration and barrier function is essential for managing skin conditions such as dryness, dermatitis, and aging [17]. 

This research article investigates the development of a *C. odorata* essential oil NE for topical treatment of scalp psoriasis and dandruff. The study focuses on various aspects such as the solubility of *C. odorata* oil, the NE formulation, particle size analysis, rheological characteristics, pH and conductivity measurements, stability tests, skin irritation, skin sebum, moisture and erythema measurements, the DPPH scavenging activity of the nanoemulgel, in vitro release and permeation studies, as well as ex vivo drug studies on permeation. In this study, we introduce a pioneering approach by formulating a nanoemulgel containing *C. odorata* essential oil for the targeted treatment of scalp psoriasis and dandruff. The novelty of our research lies in the synergistic combination of the unique properties of *C. odorata* essential oil with the advanced delivery system of a nanoemulgel, offering enhanced efficacy and prolonged therapeutic effects. 

## 2. Results and Discussion

### 2.1. Phytochemical Screening of C. odorata Oil

The findings of the phytochemical screening revealed an array of primary and secondary metabolites, as depicted in Table 1. Specifically, these encompassed alkaloids, anthraquinone glycosides, carbohydrates, flavonoids, mucilage, phenolic compounds, tannins, and volatile oils. 

### 2.2. Pseudoternary Phase Behavior of Nanoemulsion (NE)

**Figure 1:**
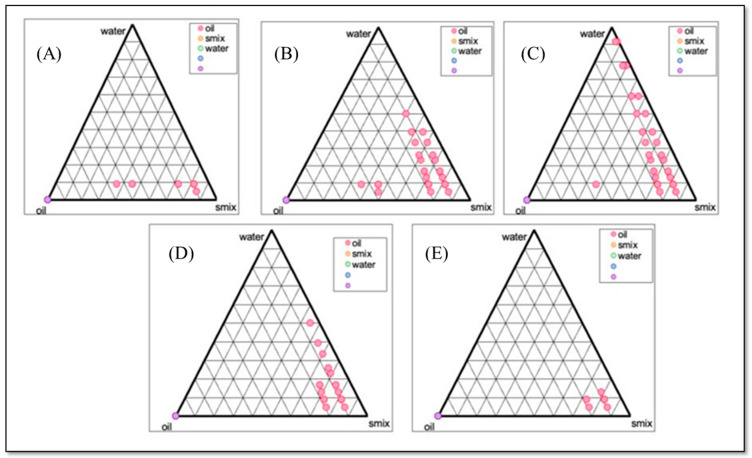
Pseudoternary phase diagrams illustrating the NE region as a function of S_mix_ composition: (**A**) 1:3, (**B**) 2:3, (**C**) 3:3, (**D**) 1:6 (selected ratio for S_mix_), and (**E**) 2:6.

Phase diagrams illustrate translucent areas where NEs exist. The remaining sections of phase diagrams are discerned through traditional and turbid visual observations. In this study, to delineate the O/W nanoemulsion regions and optimize formulations, distinct ratio diagrams for surfactant to cosurfactant in the pseudoternary phase were developed, as depicted in Figure 1.

### 2.3. Characterization Study

**Figure 2:**
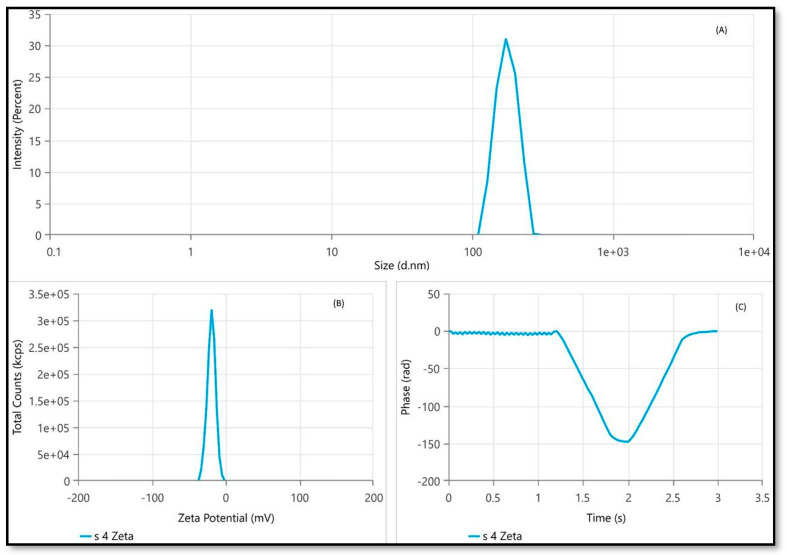
Dynamic light scattering results. (**A**) Size distribution by intensity graph of *C. odorata* NE formulation. The graph shows that the Z-average was 191.7 nm. (**B**) Zeta potential distribution of *C. odorata* NE formulation showing the mean zeta potential as –19.35 mV. (**C**) Phase vs. time graph of *C. odorata* NE.

Our characterization study revealed that the average size of the droplets was 191.7 nm, the PDI was 0.1308, and the zeta potential was −19.35 mV (Figure 2). Meanwhile, the NEs that were prepared showed droplet sizes of smaller than 200 nm for the dispersed oil phase, PDI values below 0.3, and a zeta potential (ζ) below −20 mV. These characteristics indicated kinetic stability and that the NEs were suitable for topical applications. 

### 2.4. Morphology of NE

**Figure 3:**
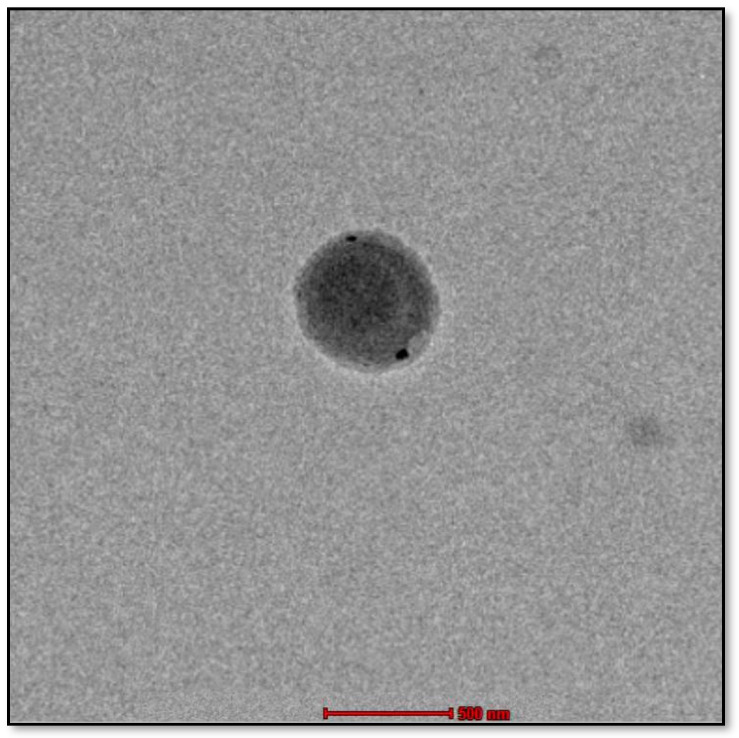
TEM image of *C. odorata* essential oil NE.

Figure 3 shows a TEM image of the *C. odorata* essential oil NE. The image shows that the diameter of the NE is 115 nm. This is in line with the hydrodynamic diameter results obtained from dynamic light scattering.

### 2.5. Physical Stability Tests and Rheological Parameters

Table 2 demonstrates that the droplet sizes, PDIs, and zeta potential values of the selected NEs exhibited no statistically significant alterations over the six-month period in various storage environments. The NE formulation has a low viscosity and a milky appearance. The viscosity of the NE at various time points and temperatures is summarized in Table 3. After a 6-month physical stability study, all selected NE formulations maintained a homogeneous state and displayed no observable physical or phase changes across the storage temperatures. 

Throughout the entire test duration, the droplet size of the NEs remained consistently between 172 ± 2.13 and 192 ± 0.50 nm. The NEs also exhibited low PDI values (below 0.2), therefore exhibiting a small droplet size distribution and high system stability. The zeta potential of the selected NEs only exhibited minimal changes, ranging from −18.36 to −19.67 mV during the three-month physical stability study. This suggests that different storage temperatures will not significantly impact the repulsive forces between NE droplets. 

The pH values of the NEs, at different time points, fell within the skin pH range of 4–6, indicating their suitability for safe application on human skin. The selected NEs demonstrated negligible pH changes under various storage conditions, affirming their compatibility for topical use.

Conductivity measurement is a valuable technique for assessing the emulsion type and detecting any variations. In line with this, the conductivity measurement results indicated no significant variations in electrical conductivity for any of the NEs across the three different stability environments over the six-month period. Higher conductivity values observed in the NEs suggest that the external phase of the NEs is aqueous, consistent with oil-in-water emulsions.

Overall, the physical stability study revealed that the selected NEs maintained their homogeneous state without significant changes in droplet size, PDI, zeta potential, pH, or conductivity, indicating their stability and suitability for topical application.

At 5 °C, the nanoemulsion exhibited consistent physical stability over time, with minimal variations observed in droplet size and PDI. Specifically, after 180 min (T180), the droplet size remained relatively constant at approximately 184 nm, indicative of uniform particle distribution. However, a slight increase in PDI from 0.1350 to 0.1840 was noted, suggesting a slight increase in particle size heterogeneity, although still within an acceptable range. Zeta potential remained negative throughout the study period, indicating sufficient electrostatic repulsion to prevent particle aggregation. The pH and conductivity values also remained stable, indicating maintenance of the emulsion’s chemical integrity. Similarly, at 25 °C, the nanoemulsion displayed stable characteristics over time, with droplet size and PDI showing negligible changes. Notably, zeta potential values remained consistently negative, ensuring the colloidal stability of the nanoemulsion. However, at 40 °C, slight variations in droplet size and PDI were observed over time, albeit within acceptable limits. Despite these changes, zeta potential values remained negative, indicating adequate repulsion forces to prevent particle aggregation.

### 2.6. In Vivo Studies

The NE formulation under examination demonstrated no observable skin irritation during the patch test conducted at the conclusion of the 48 h period. This suggests that the tested formulation was well received and adhered to safety protocols. 

To examine how the formulation impacted skin pH, pH readings were collected from volunteers’ application sites both before the test and after six weeks of product usage. The findings showed no notable disparity in the skin’s pH levels before and after the four-week application of the NE formulation under scrutiny.

At the beginning of the study, the rats with seborrheic dermatitis (SD) exhibited low skin moisture and a high sebum content. However, after applying the tested water-based formula for six weeks, the mean skin hydration index significantly increased (18.09 ± 15.07 AU to 41.28 ± 9.29), while the mean skin sebum content significantly decreased (26.75 ± 14.05 to 5.67 ± 8.75) (Table 4). The rise in skin moisture levels coincided with a reduction in skin redness. Following four weeks of using the test formulation, a statistically significant decrease in the average skin redness index was observed compared to the initial measurement (T0) (580 ± 39.09 to 520.32 ± 21.43).

### 2.7. Dermatokinetic Study

**Figure 4:**
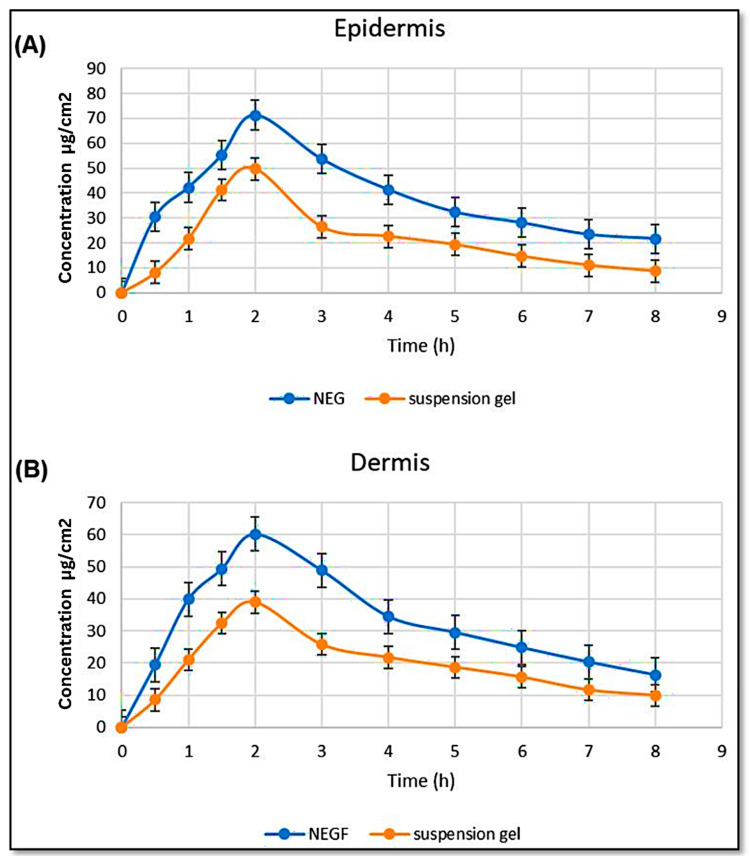
*C. odorata* nanoemulgel concentration in the (**A**) epidermis and (**B**) dermis.

The analysis of the amount of *C. odorata* present in the epidermis and dermis of rat skin after applying a nanoemulgel formulation at different time intervals was conducted using ANOVA for statistical comparison. The findings revealed that the formulation had the highest maximum concentration (C_skin max_) of 71.266 µg/cm^2^ in the epidermis, while the conventional medicine exhibited a C_skin max_ of 49.799 µg/cm^2^, which is a significantly lower concentration. On the same grounds, the NE formulation exhibited a higher C_skin max_ of 60.179 µg/cm^2^ in the dermis compared to the conventional formulation, which showed a C_skin max_ of 38.947 µg/cm^2^. Figure 4 depicts the effects of *C. odorata* NE on the concentrations in the epidermis and dermis, respectively. 

### 2.8. CCRD Studies

**Figure 5:**
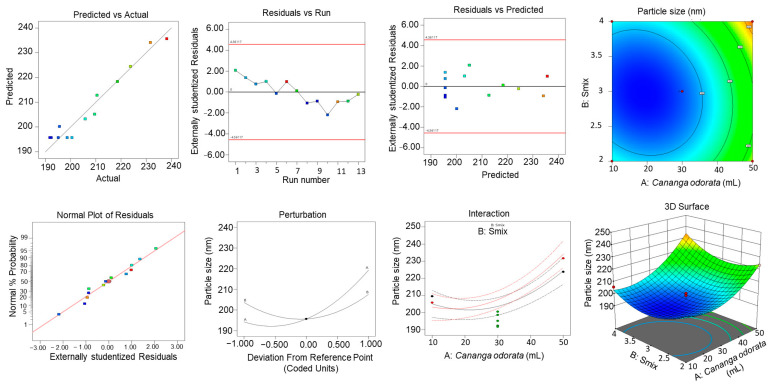
Three-dimensional response surface graphs exhibiting the effect of oil and S_mix_ concentration on particle size.

**Figure 6:**
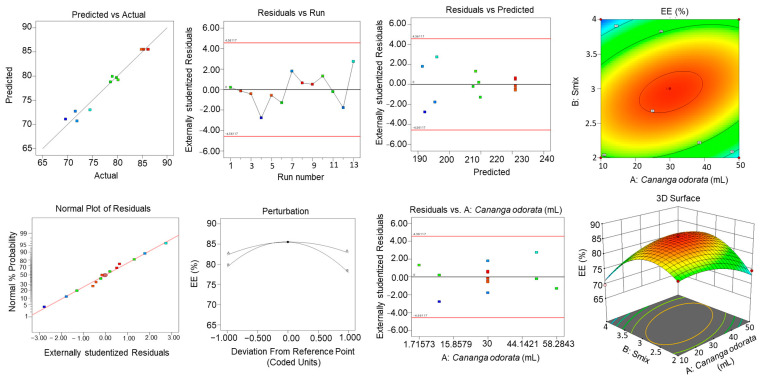
Three-dimensional response surface graphs exhibiting the effect of oil and S_mix_ concentration on zeta potential.

**Figure 7:**
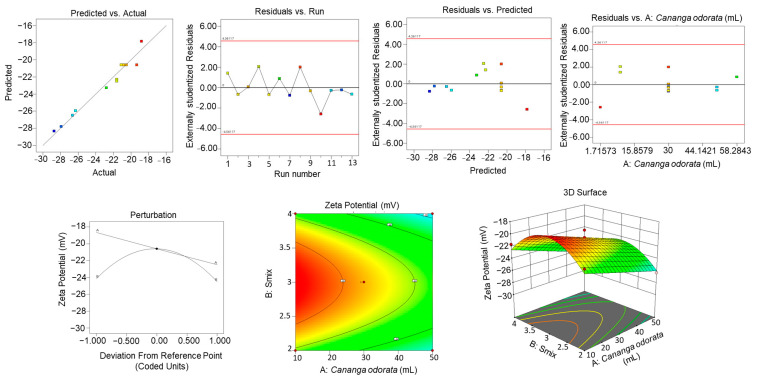
Three-dimensional response surface graphs exhibiting the effect of oil and S_mix_ concentration on entrapment efficiency.

Central composite rotatable design was employed for the optimization of the NE formulation [24]. Three-dimensional response surface graphs were constructed to show the effect of *C. odorata* oil and S_mix_ concentration on particle size, entrapment efficiency, and zeta potential (Figure 5, Figure 6 and Figure 7). Table 5 shows the responses obtained using CCRD for the particle size, entrapment efficiency, and zeta potential. 

Table 6a–c show the ANOVA results for the quadratic model, recording the sum of squares, F-value, mean square, and *p*-value. 

The final equation obtained was as follows:

Particle Size (nm) = +195.64 + 12.56A + 1.96B + 2.90AB + 11.12A^2^ + 9.95B^2^

Zeta Potential = −20.59 − 1.92A − 0.1882B − 0.0800AB + 0.0225A^2^ − 3.74B^2^

Entrapment Efficiency = +85.51 + 0.2592A − 0.7064B + 3.59AB − 2.97A^2^ − 6.90B^2^

Table 6a shows the response for particle size. The model F-value of 30.46 implies that the model is significant. The lack of fit F-value of 1.45 implies that the lack of fit is not significant relative to the pure error. There is a 35.35% chance that a lack of fit F-value this large could occur due to noise. Table 6b shows the response for zeta potential. The model F-value of 37.31 implies that the model is significant. The lack of Fit F-value of 1.66 implies that the lack of fit is not significant relative to the pure error. There is a 31.19% chance that a lack of fit F-value this large could occur due to noise. Table 6c shows the response for entrapment efficiency. The model F-value of 54.46 implies that the model is significant. The lack of fit F-value of 7.47 implies that the lack of fit is significant. There is only a 4.08% chance that a lack of fit F-value this large could occur due to noise. 

### 2.9. Effect of Variables on Particle Size

As depicted in Figure 5, as the concentration of *C. odorata* increases, the predicted particle size also increases, as indicated by the upward slope of the surface plot in the direction of the concentration of *C. odorata* axis. The colors on the surface seem to represent different ranges of particle size, with blue being the smallest size, green being medium, and yellow being the largest size within the modeled range. There are two points marked on the plot, one red and one blue, labeled as representing actual observed data that are being compared to the predicted values from the model. The red point suggests an observed particle size that is larger than that predicted by the model at that specific combination of concentration of *C. odorata* and S_mix_, while the blue point indicates an observed particle size smaller than the model’s prediction. 

### 2.10. Effect of Variables on EE%

As observed in Figure 6, there is an upward slope as the concentration of *C. odorata* oil increases, which suggests that the EE% percentage rises with higher amounts of *C. odorata* oil. Similarly, as the concentration of S_mix_ increases, there is also an upward slope, indicating that S_mix_ positively affects the EE% as well. The surface shows a peak, which represents the optimal combination of S_mix_ and *C. odorata* oil for the maximum EE%. This optimal point appears to be at an intermediate level of both S_mix_ and *C. odorata* oil, as the EE% starts to decline if either S_mix_ or *C. odorata* oil is increased beyond a certain point. 

### 2.11. Effect of Variables on Zeta Size

As shown in Figure 7, the zeta potential decreases as the levels of *C. odorata* increase, as shown by the downward slope of the surface when moving along the A axis. Likewise, an increase in the S_mix_ variable also leads to a decrease in the zeta potential, indicated by the downward slope along the B axis. The color coding suggests the magnitude of the zeta potential, with red indicating higher zeta potential values and green indicating lower values. The surface plot shows that there is a non-linear relationship between the independent variables and the zeta potential. The curvature of the surface indicates that the effect of each variable on the zeta potential may change depending on the level of the other variable; this is an interaction effect. 

### 2.12. Depth of Permeation

**Figure 8:**
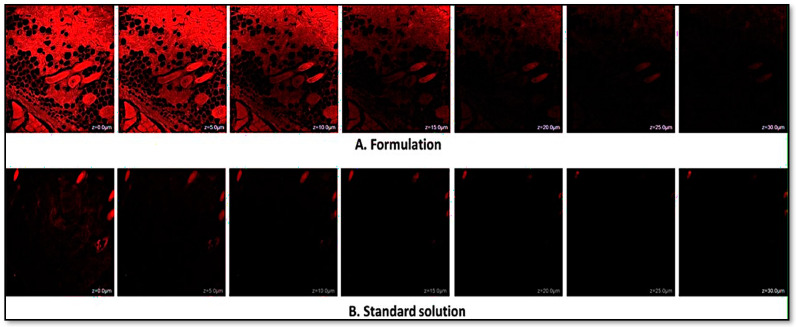
Confocal laser scanning microscopy images of rat skin under treatment (**A**) with NE gel of *C. odorata*, or (**B**) with conventional formulation. The red color depicts the deposition of the drug in the skin layers. The scale bar is equal to 10 µm.

Figure 8 depicts a comparative examination of cross-sectional CLSM images illustrating the skin surface of rats. The CLSM images assess the penetration and distribution of an NE gel in comparison to a standard formulation gel, using Rhodamine dye as a fluorescence marker within the epidermal and dermal layers of rat skin. The analysis reveals a pronounced contrast in dye dispersion patterns between the two gels. Skin treated with the standard gel predominantly shows dye accumulation at a shallow depth of 0–5 µm, indicating its residence largely on the surface, within the stratum corneum layer. In contrast, treatment with the NE gel has resulted in a more significant and deeper penetration of the dye within the skin layers. The most intense presence of dye in the NE gel-treated skin can be observed at depths of up to 30 µm, underlining its effective delivery and extended retention within the skin, beneficial for topical application. The fluorescence intensity sharply decreases past the 40 µm depth, indicating limited transdermal migration into the systemic circulation, which could contribute to localized treatment effects and reduced systemic exposure. 

### 2.13. In Vitro Release Studies

**Figure 9:**
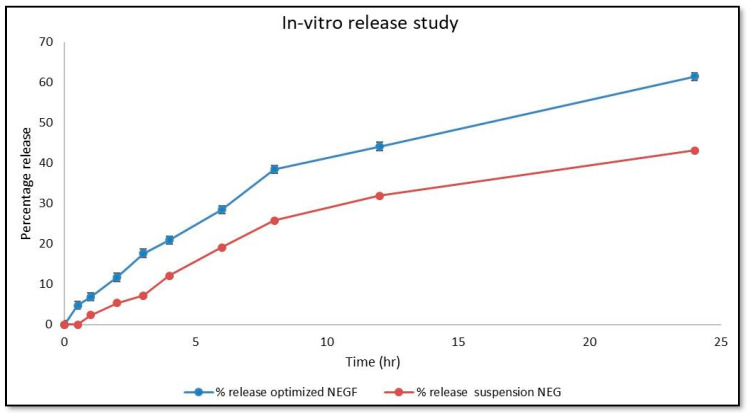
In vitro release study of *C. odorata* NE vs. conventional formulation.

The nanoemulgel formulation was compared to the conventional formulation to observe the amount of drug released in the top layer of the skin. The results of the in vitro release study for the optimized NE and its suspension are shown in Figure 9. The optimized NE exhibited a drug release percentage greater than 61.475, while the suspension had a release percentage of 43.168. The *C. odorata* nanoemulgel demonstrated a higher drug release percentage compared to the conventional formulation, and this percentage increased consistently over time. 

**Figure 10:**
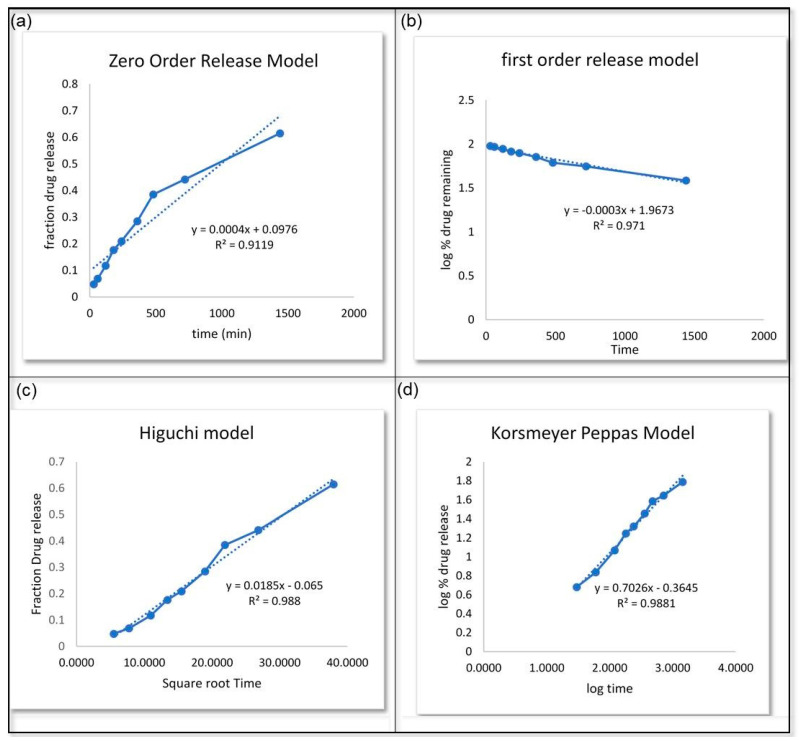
Various models were applied for the analysis of the in vitro release of *C. odorata*: (**a**) zero-order release model, (**b**) first-order release model, (**c**) Higuchi model, (**d**) Korsmeyer Peppas model.

Different models were used to analyze the in vitro drug release. Figure 10a depicts the zero-order release model, which shows a higher effect that increases over time. The graph was plotted using a y value of 0.0004x + 0.0976, and the R^2^ value was 0.9119. Figure 10b depicts the first-order release model. It was plotted with a y value of −0.0003x + 1.9673 and an R^2^ value of 0.971. This model resulted in a decrease in the logarithmic percentage of the remaining drug over time. Figure 10c displays the Higuchi model, which gave the linear equation y = 0.0185x + 0.065 and an R^2^ value of 0.988. According to this analysis, the fraction of drug release increased with time. Finally, Figure 10d illustrates the implementation of the Korsmeyer Peppas model, which gave the equation as y = 0.7026x + 0.3645 and an R^2^ value of 0.9881. The *C. odorata* nanoemulgel exhibited drug release behavior consistent with the Korsmeyer Peppas model, displaying notable drug release efficacy over time. Among the models considered, the Higuchi model was deemed optimal, while the Korsmeyer Peppas model exhibited the most robust diffusion characteristics. 

### 2.14. DPPH Scavenging Activity of C. odorata NE

Table 7 presents the mean percentages of antioxidant scavenging activity alongside the corresponding standard deviations for two different sample types at varying concentrations. For ascorbic acid, the mean antioxidant activity percentages at 10, 30, and 50 micrograms per milliliter concentrations were 86.63, 88.85, and 92.30, with standard deviations of 0.492, 0.116, and 0.061, respectively. Conversely, *C. odorata* NE showed lower mean antioxidant activities of 8.14, 14.29, and 47.14 with standard deviations of 0.136, 0.047, and 0.269 at the same respective concentrations. These results quantitatively express the efficacy of each sample’s ability to neutralize free radicals, with the standard deviation reflecting the variability of the trials conducted. 

### 2.15. Antibacterial Activity

#### Minimum Inhibition Concentration and Minimum Bactericidal Concentration

**Figure 11:**
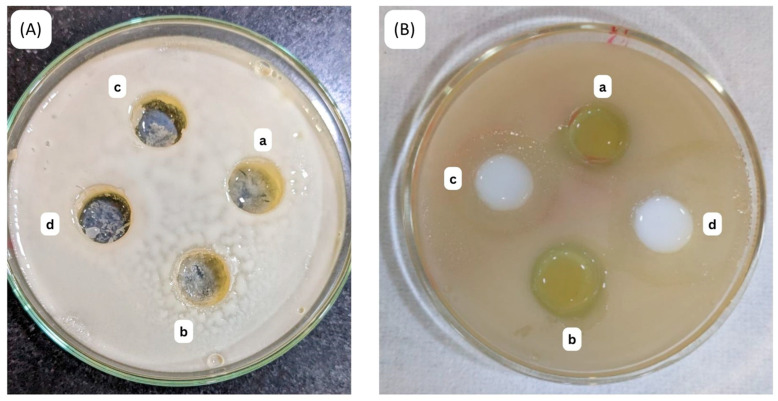
Petri dishes showing antibacterial activity against (**A**) *E. coli* for (a) *C. odorata* nanoemulgel 10 µg/mL, (b) *C. odorata* nanoemulgel 30 µg/mL, (c) ciprofloxacin, (d) *C. odorata* nanoemulgel 50 µg/mL; (**B**) *S. aureus* for (a) *C. odorata* nanoemulgel 10 µg/mL, (b) *C. odorata* nanoemulgel 30 µg/mL, (c) ciprofloxacin, (d) *C. odorata* nanoemulgel 50 µg/mL.

Table 8 and Figure 11 presents the MIC of *C. odorata* NE and ciprofloxacin against two different strains of bacteria: *E. coli* (Gram-negative) (Figure 11A) and *S. aureus* (Gram-positive) (Figure 11B). The MIC represents the lowest concentration of a substance required to inhibit the visible growth of a microorganism. In this study, various formulations of *C. odorata*, including hydroethanolic extract, oil, and NE, were tested at concentrations of 10, 30, and 50 µg/mL. Additionally, ciprofloxacin, a commonly used antibiotic, was included as a reference. For *E. coli* inhibition, the hydroethanolic extract exhibited an inhibition zone of 7.16 ± 0.17 mm at 10 µg/mL, while the oil and NE showed significantly higher inhibition zones of 11.87 ± 0.19 mm and 15.18 ± 0.87 mm, respectively, at the same concentration. As the concentration of the NE increased to 30 µg/mL and 50 µg/mL, the inhibition zones further increased to 16.48 ± 0.23 mm and 21.32 ± 0.21 mm, respectively, surpassing the efficacy of ciprofloxacin (18.65 ± 0.49 mm at 250 µg/mL). Similarly, for *S. aureus*, the NE formulations demonstrated superior inhibitory effects compared to the hydroethanolic extract and oil. At 10 µg/mL, the NE exhibited an inhibition zone of 16.15 ± 0.24 mm, which increased to 18.23 ± 0.46 mm and 23.41 ± 0.72 mm at concentrations of 30 µg/mL and 50 µg/mL, respectively. Specifically, the highest concentration of the NE (50 µg/mL) showed the highest inhibition zone, even surpassing the efficacy of ciprofloxacin (19.76 ± 0.21mm at 250 µg/mL). The MBC of the prepared nanoformulation was observed to be 50 µg/mL.

Table 9 outlines the MIC activity of *C. odorata* NE and fluconazole against two types of fungi: *Candida* and *Aspergillus*. The MIC represents the lowest concentration of a substance required to inhibit the visible growth of a microorganism. In this study, various formulations of *C. odorata*, including hydroethanolic extract, oil, and NE, were tested at concentrations of 10, 30, and 50 μg/mL Additionally, fluconazole, a commonly used antifungal medication, was included as a reference. For *Candida* inhibition, the hydroethanolic extract exhibited an MIC of 2.16 ± 0.14 mm at 10 μg/mL, while the oil and NE showed slightly higher MIC values of 3.16 ± 0.13 mm and 4.34 ± 0.46 mm, respectively, at the same concentration. As the concentration of the NE increased to 30 μg/mL and 50 μg/mL, the MIC values further increased to 5.48 ± 0.72 mm and 6.92 ± 0.22 mm, respectively. However, fluconazole demonstrated significantly higher efficacy with an MIC of 10.73 ± 0.40 mm at 150 μg/mL, surpassing the inhibitory effect of *C. odorata* formulations. Similarly, for *Aspergillus*, the NE formulations exhibited higher MIC values compared to fluconazole. At 10 μg/mL, the hydroethanolic extract, oil, and NE showed MIC values of 2.14 ± 0.54 mm, 3.79 ± 0.41 mm, and 4.12 ± 0.74 mm, respectively. With an increasing concentration, the NE demonstrated improved efficacy, reaching MIC values of 5.43 ± 0.87 mm at 50 μg/mL. However, fluconazole remained more potent with an MIC of 9.42 ± 0.27 mm at 150 μg/mL. The MFC of the prepared nanoformulation was observed to be 50 µg/mL. 

Contrary to previous studies reporting the presence of saponin in *C. odorata*, our current investigation did not validate this claim. The substantial presence of flavonoids and phenolic compounds in this plant holds particular significance for antioxidant activity. Furthermore, the identified alkaloids in our study contribute significantly to antimicrobial activity. Additionally, both flavonoids and phenolic compounds demonstrate notable anti-inflammatory and anti-cancer properties. Importantly, earlier research on *C. odorata* highlighted diverse activities, including antibiofilm, anti-diabetic, and anti-vector activities, and various medicinal properties [25]. The characterization studies provided valuable insights into the properties of the prepared *C. odorata* NEs and their suitability for topical applications. The observed results can be attributed to several factors. 

The emphasis on an NE as the chosen formulation aligns with both manufacturing feasibility and superior pharmacological actions when compared to other dosage forms. NEs offer notable advantages in terms of scalability, ease of production, and potential for large-scale manufacturing due to their simple and reproducible preparation methods [26]. The formulation’s inherent stability and extended shelf life further contribute to its industrial feasibility, making it a practical choice for mass production. From a pharmacological perspective, the NE’s fine droplet size enhances drug solubility and bioavailability, leading to improved therapeutic outcomes [27]. This is particularly crucial for essential oil delivery in scalp conditions like psoriasis and dandruff, where efficient penetration into affected areas is paramount [28]. The NE’s increased surface area allows for enhanced interaction with the scalp, optimizing the pharmacological action of the essential oil and potentially surpassing the efficacy of conventional dosage forms [29].

The average droplet size of 191.7± 1.09 nm suggests that the emulsification process successfully created fine droplets in the NEs. This result can be attributed to the appropriate choice of emulsification technique and optimized formulation parameters. The low PDI value of 0.1308 indicates a narrow droplet size distribution within the NEs. A low PDI suggests that the majority of droplets have similar sizes, contributing to the overall homogeneity of the formulation [30]. This result implies that the emulsification process was effective in producing a consistent droplet size distribution. The choice of appropriate emulsifiers and optimization of formulation parameters can help minimize variations in droplet size, leading to a low PDI value. The zeta potential measurement of −19.35 mV indicates the presence of sufficient surface charge on the NE droplets. A high absolute zeta potential value (in this case, a value close to −20 mV) suggests a strong electrostatic repulsion between droplets, preventing their aggregation and enhancing the stability of the NEs. In addition, the TEM image of the *C. odorata* essential oil NE revealed a diameter of 115 nm, consistent with the hydrodynamic diameter obtained from dynamic light scattering analysis. This congruence between the two measurement techniques provides confidence in the accuracy of the characterization results. The TEM image provides visual confirmation of the droplet size and supports the reliability of the dynamic light scattering technique in determining the hydrodynamic diameter.

The low viscosity and transparent appearance of the NE formulation indicate its ease of spreading and application [31]. These attributes are favorable for topical formulations, as they ensure good coverage and absorption upon application to the skin. The physical stability study conducted over a three-month period demonstrated the ability of the selected NE formulations to maintain their homogeneous state without observable changes in droplet size, PDI, zeta potential, pH, or conductivity across various storage temperatures. Several factors can account for the stability of the NEs over time. The selection of appropriate emulsifiers with good emulsion stability properties, such as high HLB (hydrophilic–lipophilic balance) values, can contribute to the long-term stability of the NEs [32]. The absence of significant changes in droplet size, PDI, zeta potential, pH, or conductivity indicates that the selected NE formulations were robust and resistant to physical and chemical changes under different storage conditions.

The skin irritation patch test conducted demonstrated that the tested NE formulation did not cause any visible skin irritation within the 48-h period. This indicates that the formulation was well tolerated and compliant with safety standards, suggesting its potential for safe use in topical applications. Similarly, pH measurement results revealed no significant difference in the skin pH index, indicating that the tested NE formulation did not disrupt the natural pH balance of the skin. This is an important finding, as maintaining the skin’s acidic pH is crucial for its barrier function and overall health [33]. The increase in skin hydration and decrease in sebum content indicate the formulation’s ability to restore the skin’s healthy state by improving the function of the epidermal barrier [34]. The inclusion of emollients and humectants in the formulation likely contributed to these positive effects. Furthermore, the reduction in skin erythema observed after four weeks of applying the test formulation suggests its potential in soothing and calming irritated skin. 

The dermatokinetic study conducted to assess the concentration of *C. odorata* in the epidermis and dermis of rat skin demonstrated the superiority of the nanoemulgel formulation over the conventional medicine in terms of delivering the active ingredient to the desired skin layers. The nanoemulgel formulation achieved higher maximum concentrations (C_skin max_) in both the epidermis and dermis compared to the conventional formulation. This enhanced penetration and distribution of the active ingredient can potentially lead to improved therapeutic outcomes. The confocal microscopic analysis further supported the enhanced delivery of the active ingredient by the NE gel. The fluorescence intensity of the drug was more pronounced in the epidermis and dermis layers of the skin treated with the NE gel compared to the conventional gel. This indicates that the NE gel facilitated deeper penetration of the drug, reaching the target skin layers beyond the surface, primarily limited to the stratum corneum layer.

The in vitro release studies demonstrated that the optimized NE formulation exhibited a higher drug release percentage than the conventional formulation. Over time, the release percentage consistently increased, indicating a sustained release profile. The application of various release models revealed that the Higuchi model best described the drug release behavior, followed by the Korsmeyer Peppas model [35,36]. These findings suggest controlled and sustained release of the active ingredient from the NE formulation, which can prolong its therapeutic effects. In the case of antibacterial activity against *E. coli* and *S. aureus*, the nanoemulgel demonstrated superior efficacy compared to the hydroethanolic extract and oil of *C. odorata*, as well as ciprofloxacin, especially at higher concentrations. The significant increase in inhibition zones with increasing nanoemulgel concentration indicates a dose-dependent response, highlighting the potential for optimizing its antibacterial activity. Similarly, the antifungal activity of *C. odorata* NE against *Candida* and *Aspergillus* exhibited dose-dependent inhibition, with higher concentrations showing improved efficacy. While the nanoemulgel formulations showed lower MIC values compared to the hydroethanolic extract and oil, they were still less potent than fluconazole, a commonly used antifungal medication. However, the ability of the nanoemulgel to inhibit fungal growth suggests its potential as an alternative or adjunctive therapy in antifungal treatment regimens.

In addition to the current study, several other studies have explored the synthesis and application of NEs for various essential oils [37]. For instance, Khan et al. (2018) developed an NE of clove oil using a high-pressure homogenization technique. The NE demonstrated improved solubility and prolonged release of the bioactive compounds present in clove oil [38]. Such studies highlight the versatility and potential of NE-based delivery systems for various essential oils, providing opportunities for enhanced solubility, stability, and targeted delivery of bioactive components. 

In summary, the results indicate that the tested *C. odorata* NE formulation has promising properties and potential for topical applications. It was well-tolerated without causing skin irritation, maintained the skin’s pH balance, improved skin hydration, reduced sebum content and erythema, and demonstrated enhanced drug delivery to deeper skin layers. The sustained release profile observed in the in vitro studies further supports its potential for providing long-lasting therapeutic effects. These findings collectively highlight the efficacy and potential of the tested NE formulation for addressing various skin conditions. 

## 3. Conclusions

Overall, the *C. odorata* NE exhibited desirable properties, including stability, skin compatibility, and enhanced drug delivery capabilities. These findings highlight its potential as an effective and targeted delivery system for *C. odorata* and its potential application in various dermatological treatments. However, further research is needed to explore its full therapeutic potential, optimize formulation parameters, and assess its long-term safety and efficacy in clinical settings. The *C. odorata* NE holds promise as a valuable tool in the development of innovative and effective topical treatments. 

## 4. Materials and Methods

### 4.1. Materials

*C. odorata* leaves were identified by a qualified botanist and were collected from the Botanical Garden of Jamia Hamdard, New Delhi, India with specimen number: BOT/DAC/2023/32. The following equipment was used: a UV-VIS spectrophotometer (Shimadzu-1700, Tokyo, Japan), rotor–stator mixer (Witeg HG-15D, Baden-Württemberg, Germany), 20 kHz Sonicator (Ultrasonics, Sonics & Materials, Inc., Newtown, CT, USA), Malvern Zetasizer Nano ZS (Malvern Panalytical Ltd., Malvern, UK), Brookfield cone and plate viscometer (Brookfield Engineering Laboratories, Inc., Middleboro, MA, USA), pH meter (Hanna Edge, Providence, RI, USA), Sebumeter^®^ SM 815 (Tokyo, Japan), Corneometer^®^ CM 825 device (Courage & Khazaka, Electronic GmbH, Köln, Germany), SnakeSkin dialysis bags (Thermo Scientific, Maharashtra, India), and FEI Tecnai G2 Spirit Twin Transmission electron microscope (FEI Company, Uniondale, NY, USA), Minneapolis. *C. odorata* oil (CAS Number: 8006-81-3), Cremophor^®^ EL (CAS Number: 61791-12-6), Di (ethylene glycol) ethyl ether (Transcutol^®^ HP, Gattefosse Corp Company, Paramus, NJ, USA) (CAS Number: 111-90-0), and Poloxamer 188 (CAS Number: 9003-11-6) were purchased from Sigma Aldrich Chemicals Private Limited, Bangalore, India. Preserved skin samples were purchased from ETSY Pvt. Ltd., Gurgaon, India. All the solvents and chemicals were of a good analytical grade. 

### 4.2. Preparation of Hydroalcoholic Extract of C. odorata

The hydroalcoholic extract was prepared following minor alterations to a previous protocol. The *C. odorata* leaf powder was soaked in a mixture of distilled water and ethanol in a 50:50 ratio within a flask and was placed on a shaker for agitation at an ambient temperature for a duration of one week. Subsequently, this mixture was subjected to filtration through Whatman filter paper, and the resulting filtrate was vaporized through a rotary evaporator to derive the hydroalcoholic extract with a brownish hue [39]. 

### 4.3. Phytochemical Screening of Hydroalcoholic Extract of C. odorata

Standard procedures for screening the phytochemicals present in the hydroalcoholic extract of *C. odorata* were performed using the previously established methods with a few minor changes. 

#### 4.3.1. Analysis of Alkaloids

A couple of drops of Dragendorff’s reagent were incorporated into 1 mL of hydroalcoholic extract of *C. odorata*. The occurrence of alkaloids was shown by a noticeable yellow precipitate [18]. 

#### 4.3.2. Analysis of Amino Acids

The analysis involved adding few drops of ninhydrin reagent to 2 mL of hydroalcoholic extract of *C. odorata* and observation of a purple hue to ensure the existence of proteins [19]. 

#### 4.3.3. Analysis of Anthraquinone Glycosides

The analysis was performed with the hydroalcoholic extract of *C. odorata* treated with Bornträger reagent to produce a vibrant color of purple from orange red [20]. 

#### 4.3.4. Analysis of Glycosides

This test involved adding 2 mL of Molisch’s reagent to 1 mL of the hydroalcoholic extract of *C. odorata* and thoroughly mixing the two components. Then, 2 mL of concentrated sulfuric acid was introduced to the solution by pouring it along the edge of the test container tube. The resultant mixture was then left undisturbed for a couple of minutes. The existence of glycosides was indicated by the emergence of a reddish-violet ring around the confluence of the two liquids [19]. 

#### 4.3.5. Analysis of Phenolic Compounds and Tannins

The lead acetate analysis involved adding 3 mL of a 10% lead acetate solution to 1 mL of the hydroalcoholic extract of *C. odorata*. The existence of phenolic chemicals was established by the observation of a white precipitate. Simultaneously, ferric chloride analysis was carried out, which involved stirring 1 mL of hydroalcoholic extract of *C. odorata* in distilled water and subsequently filtering the mixture. A little amount of FeCl_3_ was incorporated into the filter. When the appearance of blue-black precipitation or coloration was observed, this revealed that tannins were present [18]. 

#### 4.3.6. Analysis of Flavonoids

Sodium hydroxide analysis was performed by adding an adequate amount of a 20% NaOH solution to 1 mL of the hydroalcoholic extract of *C. odorata*. Upon the inclusion of hydrochloric acid (HCl), the formerly yellow color of the extract transformed into a colorless solution, indicating the existence of flavonoids. On the other hand, the ammonia test was performed by adding a little amount of a 1% NH_3_ solution to 1 mL of the hydroalcoholic extract of *C. odorata* in the test tube. Flavonoids were detected by the observation of a yellow tint [18]. 

#### 4.3.7. Analysis of Gums and Mucilage

The Ruthenium Red test was performed to assess the presence of gums and mucilage with a change in color to red and pink [21]. 

#### 4.3.8. Analysis of Proteins

A single drop of a 2% copper sulfate solution was added to 2 mL of filtrate for the biuret test. After that, an excess of potassium hydroxide pellets and 1 mL of ethanol were added. Proteins were ascertained to be present when the ethanol layer became pink [22]. 

#### 4.3.9. Analysis of Volatile Oils

The steam distillation procedure was conducted with minor adjustments to detect the condensation of volatile oils, which were then collected in conical flasks over a period of 120 min. The oil separated from the water and was subsequently extracted using a separating funnel [23]. 

### 4.4. Solubility of C. odorata Oil

The solubility of *C. odorata* oil in rhamnolipid (surfactants) and propylene glycol (cosurfactants) was examined by dissolving them in excessive quantities of these substances. To reach a state of equilibrium, the oil sample was continuously mixed for 10 min using a vortex mixer and kept at room temperature in a temperature-controlled shaker for 72 h. Afterwards, the equilibrated samples were centrifuged at 3500 rpm for 15 min. The resulting supernatant was filtered through a 0.45 µm filter membrane and then diluted with the mobile phase. The concentration of the oil was measured using a Shimadzu-1700 UV-VIS spectrophotometer, Japan at a wavelength of 260 nm. 

### 4.5. NE Formulation

NE was synthesized as previously reported [40]. Three different concentrations (1%, 5%, 10% *w/w*) were created using the ultrasonic emulsification method to prepare the oily phase. Two combinations of surfactants and cosurfactants (Cremophor^®^ EL/Transcutol^®^ HP and Poloxamer 188/Transcutol^®^ HP, Bangalore, India) with surfactant-to-cosurfactant ratios of 1:1 and 1:2 (*w/w*) were evaluated to determine a stable formulation for an NE. The stability of the formulation was ascertained through the combination of Transcutol (10% *w/w*) and Poloxamer (5% *w/w*), which were selected for this study. 

The oil phase and water phase were weighed and mixed by employing a magnetic stirrer for 10 min. The emulsion was then kept for ultrasonication (20 kHz, probe diameter 13 mm) for 30 min (50% amplitude, 30 s pulses on, 30 s pulses off). The beaker containing the emulsion was kept in a bigger-sized container filled with ice to maintain the emulsion temperature during sonication.

### 4.6. Particle Size Analysis

The average size of droplets and the polydispersity index (PDI) of the produced NEs were determined using DLS (dynamic light scattering). The light scattering measurements were conducted at room temperature using the Zetasizer Advance Range (Malvern Pananlytical, Westborough, MA, USA). If the PDI value exceeded 0.3, the samples were considered to have a polydisperse nature, indicating a wider range of droplet sizes within the NE [41]. To determine the zeta potential value of the NEs, the samples were diluted. The zeta potential value was then estimated based on the electrophoretic mobility of the oil droplets [42]. 

### 4.7. Morphology

The FEI Tecnai G2 Spirit Twin Transmission electron microscope (Minneapolis, USA) was used to capture TEM images of the *C. odorata* NEs. The accelerating voltage of the microscope was selected as 120 kV. The samples were diluted with aquadest and then 20 µL of the resulting solution was placed on a copper grid. The prepared grid was allowed to rest for 2 min, and excess liquid was removed. We stained 2% uranyl acetate on the grid and left that to dry [43]. 

### 4.8. Rheological Characteristics

The viscosity of the NEs was examined using a plate viscometer. The measurements were conducted at a temperature of 25 ± 0.5 °C. To assess the rheological properties of the NEs, graphs were created by plotting viscosity against shear rate. This analysis aimed to evaluate the rheological behavior of the NEs [44]. 

### 4.9. Conductivity and pH Measurements 

The pH of the prepared samples was measured using a pH meter, and the pH of the NEs was measured directly using a portable pH meter. First, the probe of the pH meter was directly inserted into each sample and readings were recorded, with the measurements taken at a temperature of 25 °C [45]. Then, the electrical conductivity of the NEs was recorded at the same temperature using the same device. 

### 4.10. Physical Stability Tests 

The samples were first kept in sealed MCTs (microcentrifuge tubes) at different temperatures for 6 months. The storage conditions included 25 °C with 60% relative humidity (RH), 40 °C with 75% RH, and 5 °C. The samples were analyzed to evaluate their particle size, polydispersity index (PDI), viscosity, zeta potential, conductivity, and pH at different time points. These measurements were conducted in triplicate [46]. 

### 4.11. Animal Study Design

Sixty male albino Wistar rats weighing between 210 and 240 g were obtained from the central animal house facility. Prior to the experiment, the rats were allowed to acclimatize for seven days in ventilated cages maintained at a temperature of 24 °C, a relative humidity of 65 ± 5%, and a 12 h light–dark cycle. During this period, the rats were provided with a standard pellet diet and unrestricted access to water. The experimental protocol employed in this study was approved by the Institutional Animal Ethics Committee at Jamia Hamdard, New Delhi (Protocol No. 1835). OECD guideline 404 was followed in relation to conducting acute dermal irritation studies. 

### 4.12. Skin Irritation Test

To assess any potential adverse effects of the formulation, a patch test was conducted prior to the application of the product. A quantity of 0.1 g of the test product was applied to the left ear of rats and observed for a 48-h observation period. 

After the 48-h period, the patch test material was removed, and the rat’s skin was examined for any signs of skin reactions such as itching, redness, irritation, or swelling. This evaluation aimed to determine whether the formulation had caused any adverse effects on the skin of rats [47].

### 4.13. Skin Sebum Measurement

A sebumete (Fisher Scientific GmbH, Schwerte, Germany) was employed to assess how the test formulation influenced the secretion of sebum on the skin. To conduct the measurement, the cartridge was gently pressed onto the measuring device until the countdown timer was displayed on the screen. As soon as the countdown timer appeared, the measurement recording was started by removing the cartridge. Holding the cartridge vertically on the skin within the measuring area for a duration of 30 s constituted the measurement interval. Subsequently, the cartridge was reinserted into the device, and the displayed value on the screen at the conclusion of the period was noted. This procedure was repeated three times within the application area, and the average value was computed [48]. 

### 4.14. Skin Moisture Measurement 

The moisturizing effect was tested using a Corneometer (CM 825- Skin Hydration Meter, Courage+Khazaka, Köln, Germany). The measurement method was based on the principle of the electrical capacitance method. During the measurement process, the probe of the device was brought into contact with the rat skin, and the water content value displayed on the screen was recorded one second after the contact was made. The outcomes were presented in arbitrary units (AU), with each AU thought to equate to 0.2–0.9 mg of water per gram of stratum corneum. Five distinct measurements were conducted within the application area, and an average value was computed to evaluate the skin’s water content and gauge the moisturizing impact of the test formulation [49].

### 4.15. Skin Erythema Measurement

Photometric evaluation of erythema was performed using a Mexameter (MX 18, Courage+Khazaka, Köln, Germany). The evaluation was based on the principle of remission. During the evaluation, the probe of the device contacted the skin, and the erythema value displayed on the screen was recorded one second after the contact was established. To ensure accuracy, three separate values were recorded. This process allowed for the quantification of erythema and assessment of its severity using the photometric measurement [50]. 

### 4.16. In Vitro Release Studies 

Snakeskin dialysis bags were employed to compare and analyze the in vitro release of *C. odorata* NE with the conventional formulation. The dialysis bags were activated, and 1 mL of the NE or conventional formulation was added to different bags. The dialysis bags were then immersed in 250 mL of phosphate buffer, pH 6.8 and stirred at 450 rpm using a magnetic stirrer. At specific time intervals, high-performance liquid chromatography (HPLC) was employed to assess the various formulations. The cumulative release of *C. odorata* oil from the membrane was recorded by plotting diffusion areas against time [51]. 

### 4.17. Dermatokinetic Studies

The application of the *C. odorata* oil nanoemulgel on rat skin was recorded using Franz diffusion cells (FDCs) [52]. The concentration of *C. odorata* NE was compared with the concentration of conventional gel at different time intervals (0, 0.5, 1, 1.5, 2, 3, 4, 5, 6, 7, 8 h). First, any extra formulation was removed from the skin by rinsing with saline. The skin was immersed in warm water at 60 °C for 3 min. The skin’s epidermis and dermis layers were isolated with forceps and fragmented into small segments. These segments were then immersed in 5 mL of methanol for a day to extract the *C. odorata* oil. After the removal of the skin layers, the remaining methanol solution underwent membrane filtration, and the *C. odorata* content was quantified via HPLC. The concentrations of *C. odorata* oil per square centimeter (cm^2^) in the dermis and epidermis layers were monitored over time, and the highest maximum concentration (C_skin max_) was documented [53]. 

### 4.18. Depth of Permeation

The CLSM 410 invert-based CLSM system (Zeiss, Heidelberg, Germany) was used to assess the depth and for visualization of the skin permeation of NE gel. In this evaluation, a formulation containing Rhodamine B dye (520 nm) was administered to the skin, which was placed in Franz diffusion cells. Following a 7 h duration of the skin permeation study, the skin was meticulously detached and cleansed with ethanol to eradicate any residual formulation. The cleaned skin was then used to create fixed slides, which were subsequently examined using CLSM to detect fluorescence within the various skin layers [36]. 

### 4.19. DPPH Radical Scavenging Activity of C. odorata Oil Nanoemulgel

The antioxidant potential of *C. odorata* oil nanoemulgel was evaluated using a DPPH assay with slight modifications to the standard protocol. Briefly, varying concentrations of nanoformulation (10, 30, and 50 µg/mL) dissolved in ethanol were mixed with a solution of 0.3 mM DPPH in alcohol. As a control, a standard solution of ascorbic acid was also prepared. The mixtures were incubated in darkness for half an hour, and the absorbance was measured at 518 nm. The percentage of DPPH radical scavenging was calculated using the following formula [25]:

$$\%\;\mathrm{scavenging}\;\mathrm{of}\;\mathrm{DPPH}=\frac{Absorbance\;of\;the\;control-absorbance\;of\;the\;nanoformulation}{Absorbance\;of\;the\;control}\times 100$$

### 4.20. Antimicrobial Activity

Two separate bacterial strains were used to test the antibacterial effectiveness of *C. odorata* NE. To perform this analysis, *Escherichia coli* (Gram-negative) and *Staphylococcus aureus* (Gram-positive) bacterial strains with ATCC numbers 25922 and 23923 and the fungal strains *C. albicans* with ATCC number 66027 and *Aspergillus* sp. with ATCC number 201291 were acquired from Sigma Aldrich Chemicals Private Limited, Bangalore, India. 

#### 4.20.1. Inoculation Preparation for Antibacterial Activity

The bacterial strains were cultured in Mueller–Hinton agar at 37 °C for 24 h until reaching a cell density of 0.5 McFarland (1.5 × 10^8^ CFU/mL). Subsequently, the cultured bacteria were harvested in sterile saline water (5 mL). Concurrently, a blank sample containing *C. odorata* NE was prepared by mixing NE with water and methanol in a 1:1 ratio to create a stock solution (5 mg/mL). This stock solution was further diluted with Mueller–Hinton agar to generate concentrations of 10, 30, and 50 µg/mL. Following this, sterile filter paper disks (6 mm) were saturated with the prepared NE solutions at varying concentrations. These disks were then positioned on agar plates previously inoculated with bacterial suspensions. The plates were left to incubate at room temperature for a day to allow bacterial growth and inhibition to occur. After incubation, the diameter of the inhibition zone around each disk was measured using a Vernier caliper. The experiment was repeated three times to ensure accuracy, and average values were computed for each concentration. The obtained results from *C. odorata* NE samples were compared against those of a commercially available drug, ciprofloxacin (5 µg/disc), which was employed as the positive control [38]. 

The assessment of minimum inhibitory concentrations (MICs) and minimum bactericidal concentrations (MBCs) of the *C. odorata* NE was carried out following a day of incubation to confirm the suppression of microbial growth. To achieve different concentrations of 10 µg/mL, 30 µg/mL, and 50 µg/mL, the *C. odorata* NE formulation was dissolved in 2.5 mL of ethanol and filtered through a Millipore filter onto clean 8 mm disks. Mueller–Hinton agar plates were employed to culture bacterial strains. Subsequently, the filter paper disks containing NE samples at varied concentrations were positioned onto the agar surface. The plates were then refrigerated at 4 °C for two hours before being shifted to room temperature for incubation for one day. Following incubation, inhibition zones were measured using Vernier calipers. The amount of *C. odorata* NE required to prevent bacterial growth on freshly infected agar plates was used as a parameter for determining the MBC [38].

#### 4.20.2. Antifungal Activity

The MIC values for *Candida* and *Aspergillus* were determined using broth macrodilution protocols based on CLSI reference documents M27-A3 and M38-A2, with minor adaptations. The experiments involved twofold serial dilutions in DMSO, resulting in different *C. odorata* NE concentrations. Inocula were prepared according to standard procedures, and for dermatophytes, conidia were harvested from 7-day-old potato dextrose agar cultures and adjusted to the required density. After 48 h of incubation, MICs were identified as the lowest concentrations of NE inhibiting complete growth of *Candida* and *Aspergillus* species. Fluconazole was used as a reference antifungal drug. Following MIC determination, MFC values were determined by subculturing 20 mL samples from all clear tubes and the last tube showing growth onto Sabouraud dextrose agar (SDA) Petri dishes. Incubation at 35 °C for a minimum of 3 days allowed for visible growth assessment. MFC values were defined as the lowest NE concentration with no observable growth [39].

### 4.21. Statistical Analysis

Statistical analysis was conducted using GraphPad Prism 9 software, and the results were presented as mean ± standard deviation (SD). Analysis of variance (ANOVA) was used for statistical testing of the CCRD. 

## Figures and Tables

**Table 1:** Phytochemical screening of *C. odorata*.

Phytochemical Test	*C. odorata* Hydroalcoholic Extract	Name of Test	Observation	Inference	Reference
Alkaloids	Present	Dragendorff’s Test	Yellow precipitate	Presence of alkaloids	[18]
Amino Acids	Absent	Ninhydrin Test	-	Absence of amino acids	[19]
Anthraquinone Glycosides	Present	Bornträger’s Test	Pink to red color	Presence of glycosides	[20]
Carbohydrates	Present	Molisch’s Test	Brick-red precipitate	Presence of carbohydrates	[19]
Coumarin Glycosides	Absent	Sodium Hydroxide Test	-	Absence of coumarin glycosides	[18]
Flavonoids	Present	Ammonia Test	Yellow coloration	Presence of flavonoids	[18]
Gums and Mucilage	Present	Ruthenium Red Test	Viscous consistency	Presence of mucilage	[21]
Phenolic Compounds	Present	Ferric Chloride Test	Greenish-black color	Presence of phenolic compounds	[18]
Tannins	Present	Lead Acetate Test	Brownish-green precipitate	Presence of tannins	[18]
Proteins	Absent	Biuret Test	-	Absence of proteins	[22]
Volatile Oils	Present	Steam Distillation Test	Aromatic smell	Presence of volatile oils	[23]

**Table 2:** Physical stability test of *C. odorata* essential oil NE (mean ± SD, *n* = 3).

Temperature	Time	Droplet Size (nm)	Polydispersity Index (PDI)	Zeta Potential (mV)	pH	Conductivity (µs/cm)
5 °C	T30	172 ± 2.13	0.1350 ± 0.01	−18.36 ± 0.79	4.78 ± 0.02	81.05 ± 1.34
	T60	187 ± 1.17	0.1395 ± 0.04	−19.85 ± 0.67	5.03 ± 0.01	85.06 ± 2.89
	T90	185 ± 2.09	0.1640 ± 0.05	−20.45 ± 0.89	4.95 ± 0.09	89.09 ± 1.89
	T180	184 ± 1.12	0.1840 ± 0.01	−21.14 ± 0.42	4.51 ± 0.01	88.28 ± 1.67
25 °C	T30	179 ± 2.17	0.1786 ± 0.10	−18.99 ± 1.84	5.08 ± 0.01	95.02 ± 1.07
	T60	190 ± 2.07	0.1367 ± 0.06	−19.45 ± 0.98	4.55 ± 0.02	101.78 ± 1.97
	T90	188 ± 1.90	0.1479 ± 0.05	−19.67 ± 1. 45	4.89 ± 0.03	128.05 ± 1.67
	T180	188 ± 0.30	0.1392 ± 0.20	−19.32 ± 1. 15	4.10 ± 0.08	127.15 ± 1.03
40 °C	T30	189 ± 0.79	0.1579 ± 0.01	−20.45 ± 0.73	4.78 ± 0.01	106.64 ± 1.45
	T60	183 ± 2.67	0.1802 ± 0.06	−18.96 ± 1.23	4.86 ± 0.03	109.04 ± 2.78
	T90	192 ± 0.50	0.1600 ± 0.03	−19.43 ± 0.67	5.08 ± 0.01	90.68 ± 2.35
	T180	191 ± 0.67	0.1720 ± 0.38	−18.91 ± 0.53	5.18 ± 0.23	91.13 ± 2.11

**Table 3:** Viscosity measurements of *C. odorata* essential oil NE during a 2-month period at 25 °C, 4 °C, and 40 °C and at 75% relative humidity.

Speed (rpm)	Viscosity (cP)						
	T1	T30			T60		
	25 °C	4 °C	25 °C	40 °C	4 °C	25 °C	40 °C
70	6.79 ± 0.11	6.98 ± 0.01	6.78 ± 0.19	6.39 ± 0.09	7.04 ± 0.61	6.78 ± 0.12	7.13 ± 0.12
75	6.90 ± 0.02	6.59 ± 0.9	6.39 ± 0.02	6.40 ± 0.13	6.93 ± 0.07	6.54 ± 0.98	6.57 ± 0.91
80	6.34 ± 0.01	7.01 ± 0.03	6.80 ± 0.21	6.20 ± 0.15	6.49 ± 0.02	7.06 ± 0.14	6.49 ± 0.13
85	6.56 ± 0.3	6.89 ± 0.01	6.50 ± 0.11	6.38 ± 0.16	6.29 ± 0.43	6.49 ± 0.12	6.48 ± 0.04
90	6.12 ± 0.12	6.49 ± 0.19	6.90 ± 0.13	6.56 ± 0.02	6.47 ± 0.51	7.12 ± 0.92	7.15 ± 0.65
95	6.33 ± 0.63	7.06 ± 0.21	7.09 ± 0.02	6.29 ± 0.08	6.39 ± 0.02	6.34 ± 0.71	6.73 ± 0.98
100	6.56 ± 0.09	6.91 ± 0.18	6.58 ± 0.13	6.49 ± 0.01	6.28 ± 0.92	6.48 ± 0.81	6.29 ± 0.93
105	6.92 ± 0.81	6.89 ± 0.12	7.04 ± 0.15	6.48 ± 0.03	6.45 ± 0. 83	6.70 ± 0.11	6.96 ± 0.12
110	6.76 ± 0.05	6.50 ± 0.1	6.75 ± 1.2	6.79 ± 0.19	6.38 ± 0.78	6.59 ± 0.18	6.52 ± 0.11

**Table 4:** In vivo study results of skin pH, skin sebum content, skin hydration, and skin erythema index before and after administration of the NE formulation. * AU—arbitrary units.

Parameter	Before Treatment	After Treatment	*p*-Value
Skin pH	5.5 ± 0.3	5.6 ± 0.1	Not Assessed
Skin sebum content (mg/cm^2^)	26.75 ± 14.05	5.67 ± 8.75	0.0453
Skin hydration index (AU) *	18.09 ± 15.07	41.28 ± 9.29	0.0232
Skin erythema index (AU) *	580 ± 39.09	520.32 ± 21.43	<0.0001

**Table 5:** Recorded values for the three responses (particle size, zeta potential, and entrapment efficiency).

Std.	Run	Factor 1	Factor 2	Response 1	Response 2	Response 3
		A: *C. odorata* oil	B: S_mix_	Particle Size	Zeta Potential	EE
		mL		nm	mV	%
1	1	10	2	209.5	−21.61	79.84
2	13	50	2	223.8	−26.3	74.51
3	4	10	4	205.8	−21.63	69.58
4	11	50	4	231.7	−26.64	78.61
5	10	1.71573	3	195.6	−18.81	80.14
6	6	58.2843	3	238.2	−22.8	78.99
7	12	30	1.58579	210.5	−27.92	71.54
8	7	30	4.41421	218.6	−28.73	71.9
9	2	30	3	200.5	−21.11	85.33
10	9	30	3	192.3	−20.84	86.11
11	8	30	3	191.7	−19.35	86.25
12	3	30	3	198.6	−20.53	85.01
13	5	30	3	195.1	−21.12	84.85

**Table 6:** (a) Response for particle size. (b) Response for zeta potential. (c) Response for entrapment efficiency.

Source	Sum of Squares	df	Mean Square	F-Value	*p*-Value	
(**a**) Response for particle size						
Model	2697.27	5	539.45	30.46	0.0001	Significant
A: *C. odorata* Oil	1261.16	1	1261.16	71.22	<0.0001	
B: Smix	30.64	1	30.64	1.73	0.2299	
AB	33.64	1	33.64	1.90	0.2106	
A²	860.78	1	860.78	48.61	0.0002	
B²	688.54	1	688.54	38.88	0.0004	
Residual	123.96	7	17.71			
Lack of Fit	64.61	3	21.54	1.45	0.3535	Not Significant
Pure Error	59.35	4	14.84			
Cor Total	2821.23	12				
(**b**) Response for zeta potential						
Model	128.75	5	25.75	37.31	<0.0001	Significant
A: *C. odorata*	29.42	1	29.42	42.64	0.0003	
B: Smix	0.2833	1	0.2833	0.4106	0.5421	
AB	0.0256	1	0.0256	0.0371	0.8527	
A²	0.0035	1	0.0035	0.0051	0.9450	
B²	97.18	1	97.18	140.82	<0.0001	
Residual	4.83	7	0.6901			
Lack of Fit	2.68	3	0.8919	1.66	0.3119	Not Significant
Pure Error	2.16	4	0.5388			
Cor Total	133.58	12				
(**c**) Response for entrapment efficiency						
Model	417.45	5	83.49	54.46	<0.0001	Significant
A: *C. odorata*	0.5375	1	0.5375	0.3506	0.5724	
B: Smix	3.99	1	3.99	2.60	0.1507	
AB	51.55	1	51.55	33.63	0.0007	
A²	61.54	1	61.54	40.14	0.0004	
B²	330.90	1	330.90	215.84	<0.0001	
Residual	10.73	7	1.53			
Lack of Fit	9.11	3	3.04	7.47	0.0408	Significant
Pure Error	1.63	4	0.4064			
Cor Total	428.18	12				

**Table 7:** DPPH scavenging activity of *C. odorata* nanoemulgel.

No.	Sample Type	Concentration (µg/mL)	Percentage of Antioxidant Scavenging Activity Trial 1	Percentage of Antioxidant Scavenging Activity Trial 2	Percentage of Antioxidant Scavenging Activity Trial 3	Mean ± SD
1	Ascorbic acid	10	86.12 ± 1.19	87.10 ± 1.92	86.68 ± 0.91	86.63 ± 0.492
2	Ascorbic acid	30	88.91 ± 1.06	88.93 ± 1.26	88.72 ± 0.98	88.85 ± 0.116
3	Ascorbic acid	50	92.35 ± 0.23	92.31 ± 0.54	92.23 ± 1.92	92.30 ± 0.061
4	*C. odorata* nanoemulgel	10	7.98 ± 2.93	8.21 ± 0.12	8.22 ± 1.21	8.14 ± 0.136
5	*C. odorata* nanoemulgel	30	14.24 ± 1.26	14.31 ± 2.10	14.33 ± 1.24	14.29 ± 0.047
6	*C. odorata* nanoemulgel	50	47.45 ± 0.12	46.99 ± 0.19	46.98 ± 0.27	47.14 ± 0.269

**Table 8:** Antibacterial MIC activity of the *C. odorata* nanoemulgel and ciprofloxacin.

Formulation	Concentration (µg/mL)	Inhibition Zone (mm)	
		*E. coli*	*S. aureus*
Hydroethanolic extract of *C. odorata*	10	7.16 ± 0.17	9.56 ± 0.81
*C. odorata* essential oil	10	11.87 ± 0.19	13.64 ± 0.43
*C. odorata* nanoemulgel	10	15.18 ± 0.87	16.15 ± 0.24
*C. odorata* nanoemulgel	30	16.48 ± 0.23	18.23 ± 0.46
*C. odorata* nanoemulgel	50	21.32 ± 0.21	23.41 ± 0.72
Ciprofloxacin	250	18.65 ± 0.49	19.76 ± 0.21

**Table 9:** Antifungal MIC activity of *C. odorata* NE and fluconazole.

Formulation	Concentration (µg/mL)	Inhibition Zone (mm)	
		*Candida albicans*	*Aspergillus fumigatus*
Hydroethanolic extract of *C. odorata*	10	2.16 ± 0.14	2.14 ± 0.54
*C. odorata* essential oil	10	3.16 ± 0.13	3.79 ± 0.41
*C. odorata* nanoemulgel	10	4.34 ± 0.46	4.12 ± 0.74
*C. odorata* nanoemulgel	30	5.48 ± 0.72	4.90 ± 0.31
*C. odorata* nanoemulgel	50	6.92 ± 0.22	5.43 ± 0.87
Fluconazole	150	10.73 ± 0.40	9.42 ± 0.27

## Data Availability

The data will be available from the authors upon reasonable request.
